# Mathematical Model of Interaction of Therapist and Patients with Bipolar Disorder: A Systematic Literature Review

**DOI:** 10.3390/jpm12091469

**Published:** 2022-09-07

**Authors:** Indah Nursuprianah, Nursanti Anggriani, Nuning Nuraini, Yudi Rosandi

**Affiliations:** 1 Doctoral Program of Mathematics, Faculty of Mathematics and Natural Sciences, Universitas Padjajaran, Sumedang 45363, Indonesia; 2 Department of Mathematics, Faculty of Mathematics and Natural Sciences, Universitas Padjajaran, Sumedang 45363, Indonesia; 3 Department of Mathematics, Faculty of Mathematics and Natural Sciences, Institut Teknologi Bandung, Bandung 40132, Indonesia; 4 Department of Geopysics, Faculty of Mathematics and Natural Sciences, Universitas Padjajaran, Sumedang 45363, Indonesia

**Keywords:** systematic literature review, bipolar disorder, mathematical model, interaction, therapist

## Abstract

Mood swings in patients with bipolar disorder (BD) are difficult to control and can lead to self-harm and suicide. The interaction between the therapist and BD will determine the success of therapy. The interaction model between the therapist and BD begins by reviewing the models that were previously developed using the Systematic Literature Review and Bibliometric methods. The limit of articles used was sourced from the Science Direct, Google Scholar, and Dimensions databases from 2009 to 2022. The results obtained were 67 articles out of a total of 382 articles, which were then re-selected. The results of the selection of the last articles reviewed were 52 articles. Using VOSviewer version 1.6.16, a visualization of the relationship between the quotes “model”, “therapy”, “emotions”, and “bipolar disorder” can be seen. This study also discusses the types of therapy that can be used by BD, as well as treatment innovations and the mathematical model of the therapy itself. The results of this study are expected to help further researchers to develop an interaction model between therapists and BD to improve the quality of life of BD.

## 1. Introduction

In 2018, around 792 million people in the world experienced mental disorders. Mental disorders are the leading causes of disability, illness, and death among the civilian population and the military. Most treatments for mental disorders currently available have limited efficacy, especially in mental disorders whose symptoms vary over a relatively short time scale [1]. As many as 0.6%, or about 46 million people, suffer from Bipolar Disorder. Bipolar disorder (BD) is the world’s sixth leading cause of disability [2]. According to the Ministry of Social Affairs of the Republic of Indonesia on its official website, in 2012, 11.6% of Indonesia’s population, which is around 24 million people, experienced mental disorders. Of the 24 million people, only 9% are undergoing medical treatment.

Naturally, everyone will experience an up or down mood. However, the mood changes experienced by patients with bipolar disorder are extreme and tend to be more rapid [3]. The moods of a BD person, ranging from severe depression to extreme mania and irritability, are often accompanied by difficulties in functioning in society [4]. Although patients with BD can achieve higher education than the general population, 65% of patients with BD are unemployed [5]. Patients with BD are people of talent and creative work [6] and also some of them are extraordinary individuals such as Ernest Hemingway, Charles Dickens, Tchaikovsky, Vincent Van Gogh, and Winston Churchill [7].

The symptoms of bipolar disorder and related disorders can be found in the Diagnostic and Statistical Manual of Mental Disorders, Fifth Edition (DSM-5). In the DSM-5, symptoms of bipolar disorder and related disorders such as: A new specifier “with mixed features” can be applied to bipolar I disorder, bipolar II disorder, bipolar disorder NED (not elsewhere defined, previously called “NOS”, not otherwise specified), and major depressive disorder (MDD); allow bipolar disorder and other related disorders for certain conditions; and anxiety symptoms are a specifier (called “anxious distress”) added to bipolar disorder and to depressive disorders (but are not part of the bipolar diagnostic criteria) [8].

In previous studies, the mood phenomena studied were hypomanic and depressed in type 2 BD [9,10]. The model of individual mood changes that are not treated uses the Van der Pol equation approach with negative damped harmonic oscillations [10]. With some assumptions, such as untreated BD, dysregulation of dopaminergic neurotransmission [11], dysfunction of mood stabilizer neurons [12], and abnormalities in circadian rhythm [13] play an important role in triggering BD. In addition to models of mental health, the Van der Pol equation is also used for several applied sciences that involve spring oscillations, such as in biology, seismology, electrical circuits [14], and the dynamics of the heart [15].

The study of interaction models between therapists and patients with BD is important because it is an ongoing topic, but publication is slow. It is hoped that many researchers will be more interested in this study because patients with BD usually have great potential and a high Intelegence Quotient (IQ) [16,17]. This hypothesis is useful for research with various further analyses. In addition, a person with BD who has a high IQ has a high risk of injury. This is because someone who has difficulty controlling their mood has a risk factor for psychological overstimulation as a consequence of a high IQ [17]. In previous studies, there were very few mathematical formulations that resulted in dynamic analysis between therapists and patients with BD. This can be seen from the literature review conducted in this study. Research shows that mathematical models of bipolar disorder can follow a variety of different systematic approaches, according to the type of mood episode, bipolar type, type of therapy, and family condition.

The method used in this study is Systematic Literature Review (SLR), which is a method for interpreting and evaluating previous research related to a phenomenon explicitly, systematically, and can be done by other researchers [18]. SLR was first used in the health sector, but now it has been used in other fields, such as management [19], mathematics [20] and insurance [21], information diffusion on social media [22], and information systems [23]. The way SLR is related to the mathematical model of interaction between therapists and bipolar disorder is quite interesting. The development of types of therapy and how the therapist interacts with patients with BD so that BD stabilizes more quickly became the initial motivation for this study, and we wanted to know how far research on the mathematical model of bipolar disorder has grown each year. In addition, what kinds of mathematical models of bipolar disorder have been extensively studied? With this knowledge, it is possible to find out how the therapist and patients with bipolar disorder interact and what therapies are good and interesting for future research and development. This research was conducted based on articles that discussed the mathematical model of bipolar disorder and its therapy.

The mathematical model of BD Therapy is a model that describes how BD therapy is carried out [24], as well as how therapy techniques can improve the mental stability of patients with BD [25]. The combination of one therapy and another is needed to accelerate the mental stability of BD patients. The development of symptoms in BD has given rise to various types of therapy, according to the risks faced. These dangers, such as chronic depression, can result in self-harm and sanctification [26]. To overcome the risks of self-harm and sacredness, intervention and protection are used [27] in addition to drug therapy, of course. Interventions can be carried out by therapists such as psychologists and psychiatrists to carry out therapy, while protection can be carried out by family, friends, and the community around patients with BD. Things that can be done include: building a collaborative and respectful therapeutic relationship in which the therapist seeks to understand the hopelessness of patients with BD and provide accurate empathy, designing an anti-suicide action plan, teaching various skills, maximizing the patient’s social support network, and fighting the stigma of bipolar disorder through acceptance of limitations while still striving to live life to the fullest through medication, an optimistic attitude, and meaningful long-term goals. From these various things, the goal to be achieved is to obtain a mathematical model of the interaction between the therapist and BD patients, with the type of therapy and duration of therapy, so that it can be seen what kind of interaction with the therapist makes BD patients stable faster. The results of the study are expected to provide an overview for future researchers to be able to develop a more accurate model of interaction between therapists and patients with BD.

## 2. Materials and Methods

### 2.1. Scientific Article Data

In this study, we selected and identified literature focusing on journals or proceedings on the mathematical model of BD and interactions with therapists. This material focuses on various types of therapy, such as drug therapy, psychotherapy, and other therapies. The articles used were obtained from several data sources, including Science Direct, Google Scholar, and Dimensions, and were published between 2009 and 2022 in English only. The articles in the literature are in the form of journal articles. These data sources were selected because they are easy to access. In selecting articles from Science Direct, Google Scholar, and Dimensions data sources, the keywords used were the same, namely “Mathematics Model” (MM), “Bipolar Disorder” (BD), “Interaction” (I), and “Therapist” (T). The articles obtained were collected in Mendeley, visualized using VOSviewer software version 1.6.16 is support by the Centre for Science and Technology Studies of Leiden University, and analyzed bibliometrically.

**Figure 1:**
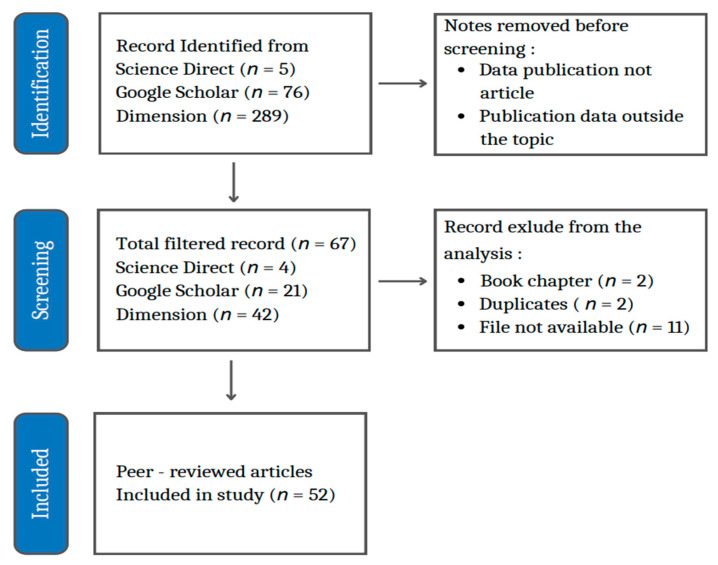
Flowchart of the search strategy.

In Table 1, it can be seen how the selection of selected articles from the data sources of Science Direct, Google Scholar, and Dimensions is done with several keywords. In the first screening process, starting with keyword 1, namely “Mathematical Models” (MM) and “Bipolar Disorder” (BD), scientific publications from Science Direct amounted to 149 articles, Dimensions 2086 articles, and Google Scholar 1960 articles. In the second screening, with the addition of the keyword “therapist” (T) and the conjunction “and”, 6 articles were obtained from Science Direct, Dimensions 305 articles, and 96 Google Scholar articles. The last screening, with the addition of the keyword “Interaction” (I) and the conjunction “and,” obtained scientific publications from Science Direct totaling 5 articles, Dimensions 289 articles, and Google Scholar 76 articles. From this last screening, the articles that can be entered into the identification, screening, and inclusion process are as listed in Figure 1.

### 2.2. Selection of Literature Database

Data for this research was obtained from three data sources (Science Direct, Google Scholar, and Dimensions), then stored in Mendeley, selected by deleting literature in the form of books or topics deemed irrelevant to this research. This selection was carried out to obtain literature that was in accordance with the purpose of this study, in the form of articles that discussed the mathematical model of the interaction between therapists and patients with BD. Each result obtained was checked one by one to get the appropriate database in the form of journal articles. Then, the articles were identified by comparing the duplicate databases from the three data sources. If a duplicate is found, the data is deleted, leaving one version of the same title in the database. The total amount of literature data obtained from the search results consisted of 382 results. After checking and sorting, 52 articles were obtained, which were included in the detailed analysis in this study. The literature review was carried out by mapping the article data obtained, including the development of research on mathematical models of therapist and BD patients’ interactions from 2009 to 2022; types of therapy in BD; interactions of therapists and patients with BD; and the development of mathematical models of BD in several countries, based on the literature used. The search process and strategies to get relevant and quality articles are given in the flowchart in Figure 1.

### 2.3. Systematic Data Analysis and Methods

The method used in this study is systematic literature review (SLR), which is an approach to obtain an overview of existing research and trends related to the mathematical model of interaction between therapists and bipolar disorder. In addition, the SLR also identifies, assesses, and interprets findings on a research topic that answer predefined research questions [28]. In general, the SLR consists of four stages, namely the determination of objectives, initiation and selection of literature, analysis, and plans to represent the results [29]. In this study, the SLR method was considered based on published articles. The articles obtained were then assessed, identified, and interpreted based on the findings obtained after reading each article, according to the research topic (i.e., therapeutic models as an alternative to BD treatment). However, in the SLR process, a systematic evaluation stage is also required when conducting research, so there is no similarity with previous research. A systematic analysis of data articles was carried out in the following stages: (1) visualization of the article database was carried out on the relationship between article data and the most discussed topics. Visualization was done using VOSviewer software to get the most used quotes; (2) mapping the number of articles each year (from 2009 to 2022), presented in the form of a bar chart, and providing general information on frequently cited research topics; and (3) mapping of the mathematical model of BD and therapists, types of therapy for BD in each country, and the number of articles that have discussed it. This mapping is done by examining the articles one by one, to determine the country and type of therapy discussed, including psychotherapy, drug therapy, and therapist interactions with clients and others.

## 3. Results

This section describes the results of data analysis focused on 52 articles, including article data visualization, development of mathematical models of BD, and types of therapy for BD. Based on the database used from three search sources, most of the references published by Scopus were 73.1%, while those cited by Scopus were 11.5%, and other references were 15.4%. The scientific work in this study was mostly published by Scopus. This can be caused because only selected scientific works are published in the form of articles in journals. In this study, unused scientific papers are in the form of theses, doctoral dissertations, book chapters, and proceedings. It is hoped that by taking articles that are mostly published by Scopus, this research can be a reference for further researchers.

### 3.1. Article Data Visualization

**Figure 2:**
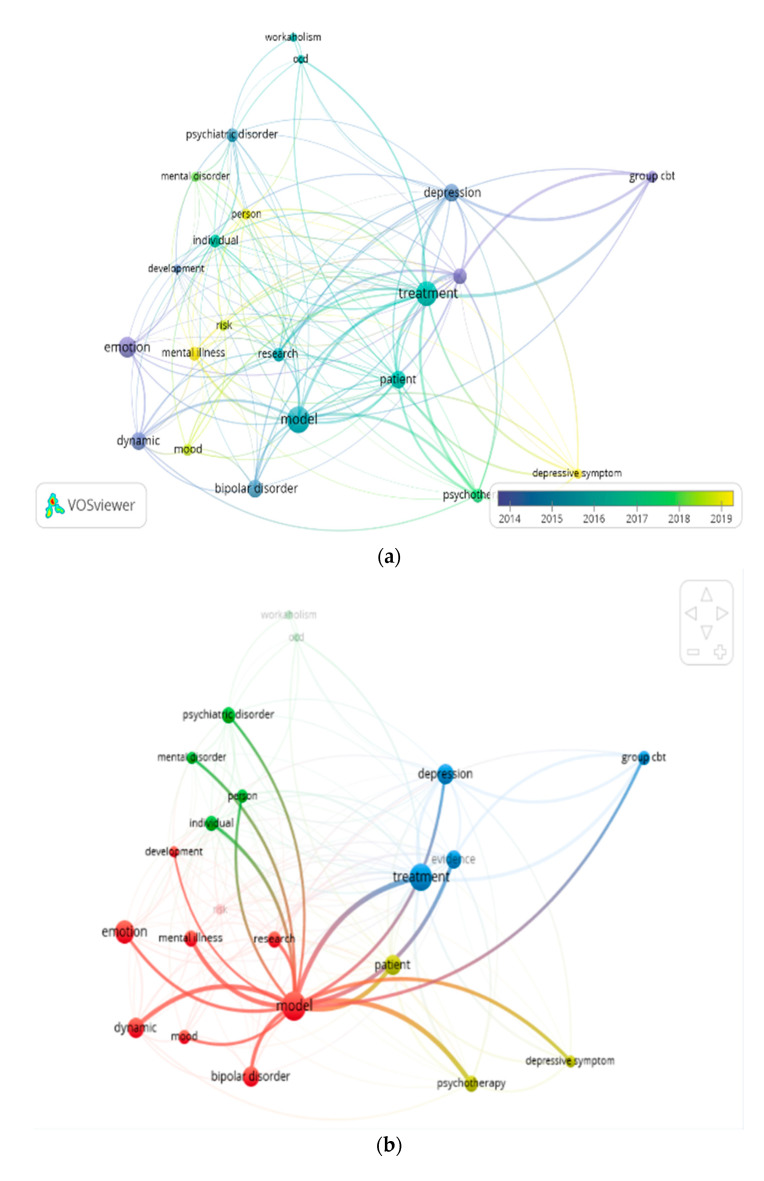
(**a**) Visualization of the article database: The aggregated network graph of the article database. (**b**) The network graph grouping the keyword “model” with other keywords from the article database.

This section provides an overview of the article data visualization obtained using the VOSviewer software. Visualization is done to find out the relationship between the data in the articles. The cluster size in the visualization shown in Figure 2 reflects how many articles cover keywords in the research topic. If the cluster in the visualization is large, it indicates that the word (quote) is present in most of the articles in the database. The line connecting the two clusters indicates that there is a relationship between the two. Furthermore, the distance between the two clusters indicates the strength of the relationship between the two clusters. For clusters that have a tendency to be strongly bound, the distance between the two clusters is shorter [30,31].

In Figure 2a, the cluster associated with the word “model” has the largest size compared to the others. This shows that the word “model” is the most talked about in the database. This word (quote) is followed by the words “emotion” and “therapy,” which rank second and third. In this picture, it can also be seen that research with the words “model” and “therapy” was mostly carried out in the 2015–2017 period, while research with the word “emotion” was carried out in 2014. Furthermore, to further determine the relationship between clusters (in this case, quote words), simply hover over the word quote that you want to see the relationship with other word quotes. From here, the relationship of the word “model” with other words is shown in Figure 2b, such as with the words “therapy”, “emotion”, “BD”, “psychotherapy”, “mental illness”, “research”, “mental disorder”, etc. From the picture, the distance between the words “model” and “therapy” is greater than “model” with “emotions.” This means that the “therapy” tendency is weaker than the “emotional” one with the “model”. Research on “models” that address the issue of “emotions” is far more than “therapy”. The quote of the word “BD”, even though it is in a small cluster, has a strong relationship with the word “model”. This means that research related to “model” and “BD” is very closely related.

### 3.2. BD Therapy Mathematical Model Development

The study of the interaction model between therapists and patients with BD is a topic that must continue because it is very much needed by the community. Despite this, publication on the topic has been slow. Although development is still slow, mathematical models of therapy in BD continue to develop around the world. The mathematical model of BD therapy has also received a good response from the government, as evidenced by the increasing support for research on mental health. The development of research shows that the mathematical model of bipolar disorder can follow a variety of different systematic approaches, according to the type of mood episode, bipolar type, type of therapy, and family conditions. In addition, it is also necessary to pay attention to research on the development of therapy for BD and combine it with the mathematical model of BD. The fact that patients with BD usually have great potential and talent as well as a high IQ [16,17] presents its own challenges for researchers so that the development of mathematical models of BD therapy can be carried out optimally so that the quality of life for BD patients increases.

**Figure 3:**
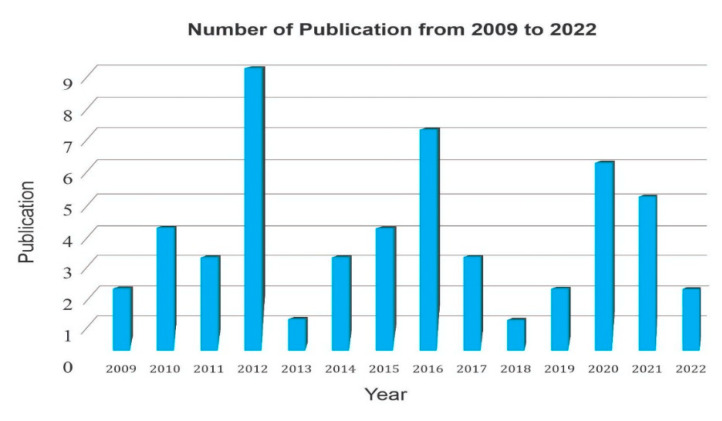
Publications from 2009 to 2022 based on the collected database.

Research conducted on the topic of the mathematical model of interaction of therapist and patients with BD has been published in three databases, namely Science Direct, Google Scholar, and Dimension. However, this research still seems slow. This indicates a novelty on this topic during the period 2009 to 2022. Research published in the form of proceedings, journal articles, and doctoral theses is collected in the literature database. However, what is reviewed in this study is only articles from journals. The development of research on the mathematical model of BD therapy from a database that is collected every year can be seen in Figure 3.

From Figure 3, it can be seen that the number of publications on the topic of BD mathematical models and therapists is still very small. The most publications were in 2012, with as many as nine scientific articles. At least in 2013 and 2018, there was one scientific article. Overall, the development of research on this topic tends to decline. This is possible because of the difficulty of research on mental health, especially BD. Therefore, it is a challenge for researchers to conduct this research. Apart from being really needed by the community, the impact of the COVID-19 pandemic has worsened the mental condition of the community.

**Figure 4:**
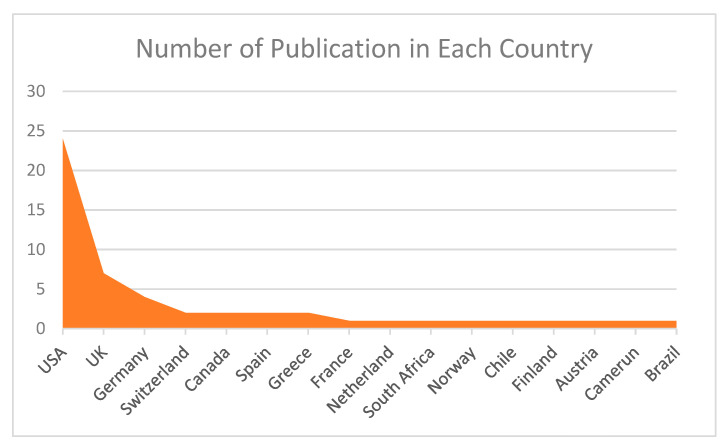
Country of publishing articles based on the collected database.

From previous research, the country that consistently discusses the topic of BD is the USA. Many scientific publications are published in that country. Based on Figure 4, the top seven countries discussing the topic of BD mathematical models and therapists are the USA, UK, Germany, Switzerland, Canada, Spain, and Greece. The countries with the most scientific publications are the USA (24 publications), the UK (up to 7 publications), Germany (up to 4 publications), Switzerland, Canada, Spain, and Greece (2 publications each). Apart from the countries above, based on the database, there are other countries that publish scientific publications on the same topic, namely France, the Netherlands, South Africa, Norway, Chile, Finland, Austria, Camerun, and Brazil (one publication each). 

**Figure 5:**
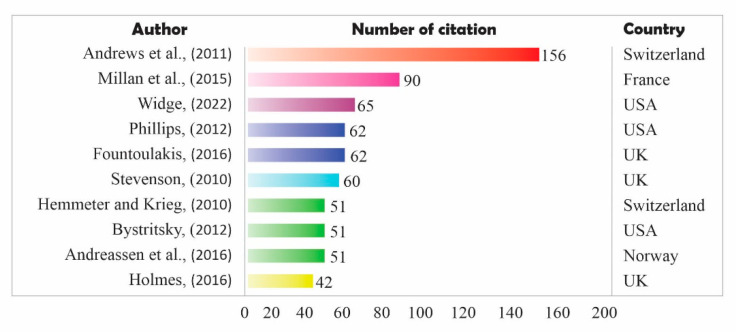
Top 10 article citations based on the collected database [1,8,32,33,34,35,36,37,38,39].

Based on the database of the previous 52 articles, the 10 articles with the most citations were classified. The number of citations shows how interesting the information provided in an article is, so that it can be used as a reference in other research articles. The more articles are cited, the more interesting the information they provide, and the more references are made to other topics related to the topic of the article in the next period. Figure 5 shows the 10 articles with the most citations, accompanied by the author and country of origin of the publisher.

Based on Figure 5, the most cited authors are Andrews et al. from Switzerland in 2011, with as many as 156 citations. This article discusses major depressive disorder, neurochemical disorders, antidepressant drug therapy, and meta-analysis of studies. In the second place, Millan et al. (2015) from France had as many as 90 citations. This article discusses neuropsychopharmacology, pharmacotherapy, therapy, and psychiatric disorders. The oldest citation was in 2010, namely Stevenson et al., from UK and Hemmeter and Krieg from Switzerland; while the latest citation is Wedge in 2022 from the USA, which is the third highest citation in the database. The fewest quotes are Holmes from UK in 2016 as many as 42 citations. Out of the 10 most-cited quotes, most of them talk about therapeutic problems in mental disorders, be it drug therapy, cognitive therapy, and others; they also discuss the diagnosis and symptoms of mental disorders; two articles discuss BD; and one article discusses computational mathematical models in psychiatry. In addition, the countries with the most citations were the USA and UK, then Switzerland, and finally France and Norway.

A detailed explanation of the research topics discussed in the top 10 articles with the most citations is given in Table 2. Of the top 10 cited articles, all of which are indexed by Scopus Q1, it can be seen that the biggest discussion of the mathematical topic of BD interaction models and therapists can be used as quotes by other researchers who want to develop this topic in the future. Based on Table 2, it can also be seen that there are two main discussions, namely therapy and psychiatric diagnosis. Of the top 10 articles, six are about therapy and four are about diagnosis. The therapies discussed can be in the form of drug therapy, cognitive therapy, and therapeutic innovation; while the diagnoses discussed were severe mental symptoms, mood disorders, mental disorders, and BD. Many (words) quotes in an article indicate that the topic is considered interesting enough to be discussed and has a great contribution for future researchers. The discussion on therapy and diagnosis in the 10 most-cited articles are explained in greater depth in the discussion section.

### 3.3. Types of Therapy in Psychiatry

**Figure 6:**
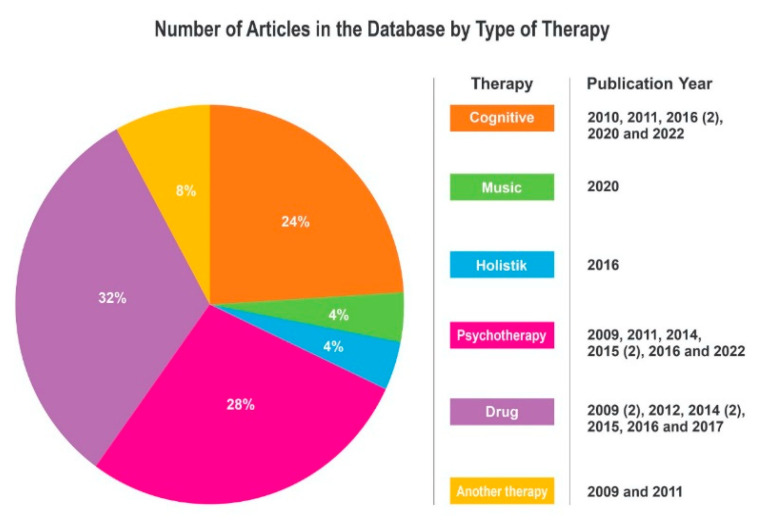
Types of therapy in the Database.

**Figure 7:**
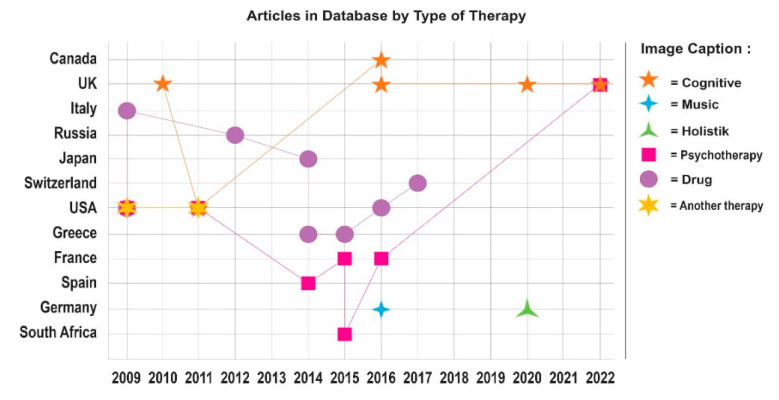
Number of articles from the database by type of therapy.

Linguistically, the meaning of the word therapy is an effort to restore the health of people who are sick or disease treatment. There are several types of therapy for psychiatric diseases, such as drug therapy, psychotherapy, cognitive, and light. The therapy is chosen to treat people with mental disorders and other psychiatric problems. Of the 52 articles selected for review in this study, there were 25 articles on therapy. The types of therapy discussed in this study can be seen in the following Ven diagram:

Based on Figure 6., of the 25 articles, it is found that the type of therapy that is widely discussed in the selected articles is drug therapy, also referred to as pharmacotherapy, in as many as 8 articles, followed by psychotherapy in as many as 7 articles. This is consistent with current practice, which is that BD treatment includes at least two types of therapy, including drugs and psychotherapy with a psychologist or psychiatrist. The next type of therapy is cognitive therapy, with six articles. Another two articles are on holistic therapy and music therapy, with one article each. From Figure 7, it can be seen that most of the articles were published in the USA (seven articles); followed by UK (five articles); France (three articles); Germany (two articles); and then Italy, Russia, Japan, Switzerland, Greece, Spain, South Africa, and Canada (one article each).

### 3.4. Methodology Used in Types of Therapy

The methodology used in this type of drug therapy research usually uses experimental methods by taking data before and after therapy is carried out. This method takes a sample of BD individuals who have been treated with drugs for some time. The time span used is usually quite long, so that the effect of the treatment can be seen for each individual sample taken. In addition, monitoring is also carried out during therapy so that psychopathological conditions can be seen [40]. The long-term effects of drug therapy are also seen, so that the possibility of changing from one drug therapy to another is still monitored by a psychiatrist.

In this type of holistic therapy, one of the methods used is the Clinical-Neuropsychological Perspective, which uses a comprehensive perspective both in terms of clinical and neuropsychological/biological neuropsychology [41]. The holistic approach views humans as a whole, in the sense of humans with cognitive, affective, and behavioral elements. With this type of therapy, humans cannot stand alone but are closely related to their environment. Treatment of brain disorders with holistic therapy must include aspects of individuality and subjectivity, treating the sequelae of brain damage in clinical neuroscience, which demands a biopsychosocial perspective, both for conceptual and historical reasons.

Several articles conducted a series of design studies for cognitive therapy, including design workshops and prototype testing in people who had previously received cognitive therapy for depression, as well as qualitative interviews and role-playing sessions with cognitive therapists experienced in depression treatment [42]. In addition, there are also those using different linear and quadratic mixed model analysis methods with random effects for each patient tested. In this method, baseline change is defined as the percentage change in individual symptoms during the first 2, 3, 4, and 5 weeks. Then symptoms from sessions four, five, six, seven, and so on were predicted using different models, with the initial changes added to the model in the last step [43].

In this type of psychotherapy, methodological and substantive issues are raised in relation to what can be said about evidence-based psychotherapy and its effects. Among the methodological problems are control conditions comparing evidence-based psychotherapy with selective reporting of measures and the lack of evidence that evidence-based psychotherapy has a significant clinical impact [44]. The success of psychotherapy depends on the nature of the therapeutic relationship between the therapist and client. In selected articles, there are those who use dynamic systems theory to model the dynamics of emotional interactions between therapists and clients, using a very similar approach (physics-science paradigm) to the model and make predictions about the relationship between therapist and client [45].

Methods for this type of music therapy examine the impact of this therapy on circadian biological rhythms in everyday life. In particular, the vagal tone circadian rhythm was monitored, indexed by heart rate variability (HRV), and the hypothalamic–pituitary–adrenal (HPA) axis, and indexed by the diurnal cortisol profile. Observations were made before and after therapy, with a treatment time span of about 10 weeks. Then, before and after the intervention period, psychological data (48 h HRV, salivary cortisol samples for 2 consecutive days), and observer ratings were collected. As a result, music therapy affects the HPA axis and autonomic regulatory processes [46].

## 4. Discussion

In some cases, mood changes occur in a certain pattern. There are several types of mood episodes that occur in bipolar disorder, including manic, depressive, hypomanic, and mixed [47,48]. To achieve a stable condition, patients with BD need to take treatment in the form of drugs and therapy regularly. Otherwise, over time it will get worse [49]. More than 20% of BD patients (mostly without treatment) end their lives by suicide [50]. It was also found that 18% of patients with BD had the urge to harm themselves at night, either using sharp weapons or poison [51]. In addition, the mental stability of patients with BD also affects the family or people living with them [52]. Mood phenomena that occur in BD need to be studied in depth. This can minimize the instability of patients with BD. The treatments that can be done for BD include psychotherapy [53], ISRT [54], food [55], and drugs [56]. With the right therapy, it will greatly help the mental stability and safety of patients with BD.

### 4.1. Research Trends in Mathematical Models of Interaction of BD and Therapists

Based on Figure 2a and Figure 3, it can be seen that research on this topic is still very small and its development has decreased. In Figure 2a, research in 2014 in the form of dark blue dots (dynamic, emotion, depression, cbt group) is still far more than the research in 2019 in the form of yellow dots (mood, mental illness, risk, person). While in Figure 3, it can be seen that the number of articles published from 2009 to 2022 decreased. This can give new researchers an opportunity to research this topic in more depth, with a higher publication value because of its high novelty. According to Siqi Xue et al. [57], the COVID-19 pandemic has posed significant challenges to healthcare globally, and individuals with BD are disproportionately affected. Individuals with BD will experience poorer physical and mental health than normal people because several risk factors associated with BD, including impaired social rhythm, risk-taking behavior, substantial medical comorbidities, and common substance use, can be exacerbated by lockdown, social isolation, and decreased preventive and maintenance care in the face of the COVID-19 pandemic. Research on this topic during a pandemic is an additional challenge and could become a new topic linking mitigation strategies for working with individuals with BD in clinical and research contexts with a focus on digital medicine strategies to improve quality and accessibility to services. It is hoped that the development of research on this topic will increase further because it is not affected by environmental conditions due to the COVID-19 pandemic.

In Figure 4, it can be seen that the development of publications on the topic of the mathematical model of the interaction of BD and therapy is dominated by the USA and European countries. This is closely related to the high number of BD sufferers in the USA and European countries, thus increasing the interest of researchers in these countries in this topic. From the data on the https://ourworldindata.org [58] website accessed on 17 July 2022, you can see a graph of the total number of patients with BD in the USA, UK, France, Switzerland, and Norway, measured for both sexes and all ages. These figures provide an accurate estimate (beyond reported diagnoses) of the number of patients with BD based on medical, epidemiological, survey, and meta-regression modeling data.

To obtain more article data, it can be done with other data sources such as Crossref, Web of Science, and Microsoft Academic. So, hopefully the analysis and findings can be more specific. In addition, articles that are not only in English but also in other languages such as Spanish, French, German, Russian, Arabic, and others, are also considered to expand the range of articles so that the research findings are more comprehensive.

### 4.2. Therapeutic Analysis and Psychiatric Diagnosis

In this section, we discuss the results of the analysis obtained from the literature review (Table 2) of the 10 articles with the top citations. The main topics covered in the 10 articles are psychiatric therapy and diagnosis.

Andrews et al. [32] described major depressive disorder (MDD) and its treatment with antidepressant drugs. Episodes of major depressive disorder (MDD) have five of the following nine symptoms: (1) depressed mood; (2) anhedonia; (3) a significant decrease (or gain) in weight or appetite; (4) insomnia (or hypersomnia); (5) psychomotor retardation (or agitation); (6) fatigue or loss of energy; (7) feelings of worthlessness or guilt; (8) reduced ability to concentrate; and (9) repeated thoughts of death (not just a fear of death), or thoughts or actions of suicide. In this article, MDD is given therapy in the form of anti-depressants, and the effect is seen on the symptoms that arise.

KN Fountoulakis et al. [34] selected treatment specifically for acute mania, mixed episodes, acute bipolar depression, maintenance phase, psychotic and mixed features, and anxiety; cycles were immediately evaluated with regard to drug efficacy. The drug therapy used for BD includes anticonvulsants, antidepressants, antipsychotics, lithium, mood stabilizers, and others, which are complemented by clinical trials of each of these drugs.

In U.-M. Hemmeter et al. [36], the acute response to sleep deprivation (SD) is an investigation of the basic neurobiological mechanisms underlying depression and antidepressant treatment. Focusing on the neurobiology of depression and the discovery of the integration of new methods in psychiatric research, such as neuroradiology and neuroendocrinology, a number of new findings have been developed on brain function in depression, which touch on the relationship between SD and depression. The therapy used is anti-depressant drugs along with sleep therapy.

The cognitive therapy discussed in the article by Holmes et al. [39] focused on a new image (MAPP; Mood Action Psychology Program) targeting mood instability and applied the measurement method in a non-concurrent multiple base design case series of BD. After that, treatment innovation was carried out with the aim of detecting an increase in BD mood stability. These innovations can be in the form of pharmacological or psychological treatments carried out together or individually.

From the several therapies above, it is concluded that drug therapy always accompanies other therapies such as sleep therapy, cognitive therapy, MAPP, and others. This is because a person with BD has a lack of neurotransmitter substances in their brain [59], so drug therapy is the main therapy and other therapies are complementary therapies. Nonetheless, cognitive behavioral therapy, family-focused therapy, and psychoeducation offer the strongest efficacy in terms of relapse prevention, while interpersonal therapy and cognitive-behavioral therapy may offer more benefit in treating residual depressive symptoms [60]. 

The most widely discussed psychiatric diagnosis in the 10 articles above is depression symptoms, which is one of the symptoms of mental disorders [61]. To diagnose symptoms of depression, you can use the DSM-5 (Diagnosis and Statistical Manual of Mental Disorders) [8]. In addition to the article Wedge et al., the diagnosis made in mental disorders is to identify biomarkers or neurological signs of mental illness and fluctuating symptoms through neuroimaging and electroencephalography (EEG) [1]. The most common diagnoses were anxiety disorders (15.8%), followed by depression (6%) and somatoform disorders (5.6%) using the Primary Care Evaluation of Mental Disorders (PRIME-MD) Patient Health Questionnaire (PHQ), or abbreviated PRIME-MD PHQ [62]. 

### 4.3. Research Development in Mathematical Models of Interaction of BD and Therapists

This research has limitations, because it only knows about which countries do the most BD research, what topics are discussed the most, the number of citations in articles, and the tendency for the number of articles to continue to decline every year especially with the COVID-19 pandemic. Although the time period taken is 24 years from 2009 to 2022, the development of this research has a decreasing trend. In this research, there was no discussion of more specific BD problems regarding response to therapy, so it is necessary to study more deeply what things must be considered so that therapy can be chosen that can make mood stability from BD occur fast.

The interesting thing that can be developed from this research is to offer a client therapist model into a more general mathematical model of the interaction between therapist and patient with BD, so that the developed model describes mood stability from BD to be stable fast.

## 5. Conclusions

In this study, a systematic literature review is presented on the mathematical model of the interaction between bipolar disorder and therapists. There were 370 articles obtained after screening scientific publications from data sources Science Direct, Dimension, and Google Scholar. After going through the selection of duplicates, book chapters, titles, and abstracts, 52 selected articles were obtained. From these 52 articles, they were then selected based on the most words (quotes), the most topics, and the countries with the most publications. There are very few publications on the topic of BD mathematical models and therapists. The most publications were in 2012, with as many as nine scientific articles. At least in 2013 and 2018, there was one scientific article. Overall, the development of research on this topic tends to decline. This is possible because of the difficulty of research on mental health, especially BD.

In this study, bibliometric analysis was also carried out on the data in Table 2, so that two main discussions were obtained, namely therapy and psychiatric diagnosis. From the data in Table 2, six articles discuss therapy and four articles diagnosis. The therapies discussed can be in the form of drug therapy, cognitive therapy, and therapeutic innovation, while the diagnoses discussed were severe mental symptoms, mood disorders, mental disorders, and BD. 

The use of bibliometric mapping techniques can reveal a general picture of existing themes and their changes over time. Based on bibliometric results, information reveals that the development of publications on the topic of the mathematical model of BD interaction and therapy is dominated by the USA and European countries. This is closely related to the high number of BD sufferers in the USA and European countries, thus increasing the interest of researchers in these countries in this topic. 

This research can show that there are very few publications on the mathematical model of the interaction between BD patients and therapists, and that the number tends to decrease every year. This has the author’s attention, as well as a new hope for this research. Nevertheless, this research is expected to continue to grow because the need for mental health knowledge is very much needed, especially during the current COVID-19 pandemic. It is hoped that with SLR and bibliometric, other researchers can see which topics are still vacant so that they can conduct more in-depth research on these topics. In addition, by knowing the topic that is the main discussion, it is hoped that future researchers can conduct research by developing appropriate therapeutic techniques and diagnosing BD so that their quality of life improves and their safety is guaranteed.

The purpose of this research is the success of therapy and to simulate the success of therapy strategies. From this literature study, it has implications for choosing the right therapeutic method, so that BD mood can stabilize fast. The best strategy will be made in determining the best combination between drugs and psychotherapy, as well as making qualitative research into quantitative ones.

## Figures and Tables

**Table 1:** Number of publications from three databases with three types of keywords.

Keywords Screening	Type	Science Direct	Dimensions	Google Scholar
Keyword 1	MM and BD	149	2086	1960
Keyword 2	MM and BD and T	6	305	96
Keyword 3	MM and BD and T and I	5	289	76

Description: Mathematical Models: MM; Bipolar Disorder: BD; Therapist: T; Interaction: I.

**Table 2:** Research topics from the top most-cited articles.

Author	Indexed	Keywords	Citation	Focus
PW Andrews, et al. (2011) [32]	Scopus Q1	Major depressive disorder, neurochemical disorder, antidepressant drug therapy, meta-analysis of studies	156	tested the prediction of revival of depressive symptoms comparable to the effect of ADM disorder by conducting a meta-analysis of ADM discontinuation studies.
MJ Millan, et al. (2015) [33]	Scopus Q1	Neuropsychopharmacology, pharmacotherapy, therapy	92	Neuropsychopharmacology enhancement increased prevention and assistance of psychiatric disorders.
AS Wedge et al. (2022) [1]	Scopus Q1	Deep brain stimulation, Psychiatric illness, Psychiatric diagnosis, Functional imaging, Anxiety disorders, Electrophysiology, Modeling, Local field potential, Mood disorders.	65	developed a closed-loop DBS system by correcting dysfunctional activity in the brain circuits underlying the domain.
J. Phillips et al. (2012) [8]	Scopus Q1	Diagnostic and Statistical Manual of Mental Disorders, diagnostic category	62	Development of DSM and DSM-5 in mental disorder and diagnostic category
KN Fountoulakis et al. (2016) [34]	Scopus Q1	Bipolar disorder; Anticonvulsants; Antidepressants; Treatments; Clinical; Clinical trials	62	systematic literature search, detailed presentation of results, and assessment of treatment options in terms of efficacy and tolerability/safety BD.
MD Stevenson et al. (2010) [35]	Scopus Q1	Group cognitive behavioral therapy, postnatal depression, clinical effectiveness, cost-effectiveness, value of information analyses	60	support the use of cognitive behavior therapy (CBT) in the treatment of depression, and psychological therapies as a first-line treatment for PND.
U.-M. Hemmeter, et al. (2010) [36]	Scopus Q1	depression, dopamine, GABA, microsleep, naps, neuroimaging, serotonin, sleep deprivation, sleep EEG, sleep endocrinoloy, sleep regulation	51	sleep deprivation problems in depressed patients and the use of antidepressant drugs as well as neurobiological aspects of sleep and SD (sleep EEG, neuroendocrinology, neurochemistry, and chronobiology.)
Bystritsky, A (2012) [37]	Scopus Q1	Phenomenology, Mathematical models, Non-linear dynamics, Winner less competition Psychopathology	51	highlights the significant potential benefits of applying computational mathematical models to the field of psychiatry, particularly in relation to diagnostic conceptualization.
CS Andreassen, et al. (2016) [38]	Scopus Q1	Workaholism, a cross-sectional survey based on the web, Symptoms of Psychiatric Disorders	51	assessing symptoms of psychiatric disorders and work addiction.
EA Holmes et al. (2016) [39]	Scopus Q1	BD, mood fluctuations, treatment innovation, MAPP; Mood Action Psychology Program	42	Innovative treatment for bipolar disorder.

## Data Availability

Not applicable.
